# Supplementary material for: Parental age selection in C. elegans influences progeny stress resistance capacity
Source: J Gerontol A Biol Sci Med Sci. 2025 Oct 30;80(12):glaf221. doi: 10.1093/gerona/glaf221 (PMC12613252; doi:10.1093/gerona/glaf221)
Supplement: glaf221_Supplementary_Data [file glaf221_supplementary_data.zip › 092925 R3 Van Camp PAS supplemental.pdf]

SUPPLEMENTAL MATERIALS

Supplemental Figures

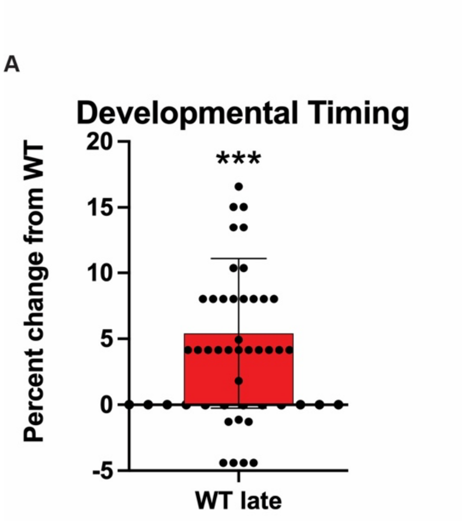

Figure S1: (**A**) Developmental timing from egg to first egg laid for WT Late worms compared to WT (N=3, n=35 total)

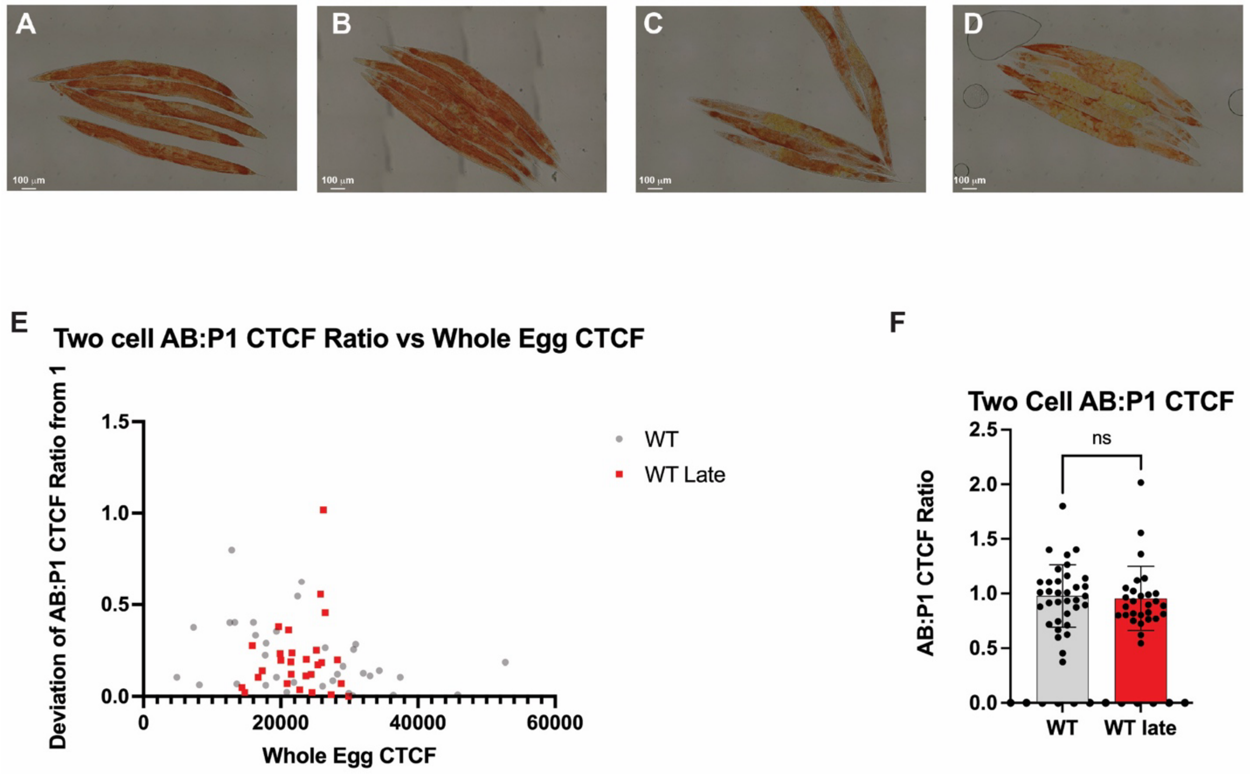

Figure S2: (**a**) WT non-Asdf worms (**b**) WT Late non-Asdf worms (**c**) WT Late intermediate Asdf worms (**d**) WT Late Asdf worms (**e**) Nile Red stained lipid distribution between AB and P_1 cells vs total lipid content of the egg in WT and WT Late worms (N=3, n=a minimum of 28 total) (**f**) Nile Red stained lipid distribution between the AB and P_1 cells of WT and WT late animals (N=3, n=a minimum of 28 total).

SUPPLEMENTARY TABLES

	WT	WT Late	Percent Difference
Quartile 1	16	11	-31%
Quartile 2	19	12	-36%
Quartile 3	21	14	-33%
Quartile 4	29	23	-20%
Median	19	12	-36%

Table S1: Quartiles for WT and WT Late lifespan assay

Supplemental xls files

Table S2: *gst-4::gfp* reporter RNAi of GWS hits results

Table S3: WT vs WT Late RNAseq DEG List
